# Integrated plasma metabolomic and proteomic analysis uncover the effects and mechanisms of isotretinoin in severe acne

**DOI:** 10.3389/fphar.2025.1590820

**Published:** 2025-08-08

**Authors:** Manqi Xia, Jing Zhang, Yang Hu, Ziyan Chen, Yanan Ke, Shujuan Zhang, Zeen Yang, Xin Tian, Jingyao Liang, Yumei Liu

**Affiliations:** ^1^ Institute of Dermatology, Guangzhou Medical University, Guangzhou, China; ^2^ Department of Dermatology, Guangzhou Dermatology Hospital, Guangzhou, China; ^3^ Department of Dermatology, Qianxi People’s Hospital, Qianxi, China

**Keywords:** acne vulgaris, isotretinoin, plasma, metabolomics, proteomics, mechanism

## Abstract

**Background:**

Acne vulgaris is a prevalent chronic inflammatory disorderof the skin and oral isotretinoin is one of the most effective treatments forsevere acne with incompletely understood mechanisms. The aim of thisstudy was to investigate the pathogenesis of acne and the therapeuticmechanisms underlying isotretinoin treatment from integrated human plasma metabolomics and proteomics.

**Methods:**

Liquid chromatography-tandem mass spectrometry (LC-MS/MS) full-spectrum metabolomics and four-dimensional data-independent acquisition (4D-DIA) quantitative proteomics were employed to analyze plasma samples from patients with group AG (severe acne group), group AG1 (severe acne group1, before isotretinoin treatment), group TG (isotretinoin treatment group) and group CG (control group). Bioinformatics statistical analysis were employed to analyze the metabolomic and proteomic data.

**Results:**

489 differentially expressed metabolites (DEMs) were detected in the plasma from patients with severe acne compared to controls. Isotretinoin treatment normalized the dysregulation of 94 metabolites, including inositol 1,3,4-trisphosphate (Ins(1,3,4)P3), 11-cis-retinol, thyroxine (T4), androstenediol, estrone 3-sulfate, bovinicacid, n-oleoylethanolamine, LPS(20:1), Cer(d16:1/23:0) and TG(17:1 18:2 18:3). Additionally, 36 differentially expressed proteins (DEPs) were identified in patients before and after isotretinoin treatment. Notably, downregulation of acyl-CoA synthetase long-chain family member 4 (ACSL4) suggests a potential therapeutic mechanism for isotretinoin, while upregulation of monoacylglycerol O-acyltransferase 2 (MOGAT2) may mediate the elevation of blood lipids and the correction of some abnormal lipids. Isotretinoin modulates multiple pathways, including inositol phosphatemetabolism, glycerolipid metabolism, thyroid hormone synthesis and insulin resistance.

**Conclusion:**

Important DEMs, DEPs and metabolic pathways were identified in this study, which will help clarify the pathogenesis of acneand the potential mechanisms of isotretinoin in the treatment of acne, andidentify novel targets for severe acne treatment and side effect reduction.

## Introduction

Acne vulgaris is a common chronic inflammatory dermatological condition affecting the pilosebaceous units, clinically manifesting as comedones, papules, pustules, nodules, and cysts. Severe acne is recurrent, painful, and often results in scarring, significantly impacting patients’ aesthetics, physical health, and psychological wellbeing (Cruz et al., 2023). The etiology of acne is multifactorial, involving sebaceous gland hyperactivity, abnormal keratinization, proliferation of *Cutibacterium acnes* (formerly *Propionibacterium acnes*), and a complex inflammatory cascade (Cruz et al., 2023). Accumulating evidence suggests a link between acne and metabolic disorders, such as elevated androgen levels, insulin resistance, and hyperlipidemia (Bungau et al., 2023). However, the pathophysiological mechanisms underlying severe acne remain incompletely understood, highlighting the need for further investigation into its potential pathogenic processes.

Isotretinoin (13-cis-retinoic acid) is a potent oral retinoid that exerts its therapeutic effects by binding to retinoid receptors. It exhibits multiple beneficial actions, such as inhibiting sebaceous gland secretion, regulating abnormal keratinization in pilosebaceous ducts, reducting *Cutibacterium acnes* proliferation, exerting anti-inflammatory actions, and preventing scar formation. Oral isotretinoin is one of the most effective treatments for severe acne, although it is associated with various adverse effects, including skin and mucous membrane dryness, elevated lipid levels, teratogenicity, and increased liver enzyme levels (Costa et al., 2018). The mechanisms by which isotretinoin affects the body are complex and not fully understood. Research has demonstrated that isotretinoin significantly alters lipid metabolism by decreasing sebum production while concurrently increasing serum lipid levels (Khabour et al., 2018). Nevertheless, the changes in plasma metabolic profiles before and after isotretinoin treatment, as well as its effects on metabolic and protein pathways, remain unclear.

Metabolomics and proteomics, as high-throughput technologies, provide powerful tools for comprehensively analysis of metabolites and proteins associated with disease states and therapeutic interventions (Wörheide et al., 2021; Nesvizhskii, 2014). Metabolomics emphasizes the qualitative and quantitative analysis of metabolites and metabolic pathways, investigating changes in metabolites and potential mechanisms in response to diseases or drug treatments (Wörheide et al., 2021). Proteomics utilizes techniques such as mass spectrometry and bioinformatics to conduct comprehensive analyses of proteins, with the aim of elucidating their composition, function, and interactions in cells, tissues, or body fluids (Nesvizhskii, 2014). The integration of metabolomics and proteomics allows for the examination of physiological and pathological activities from multiple perspectives, enabling a systematic depiction of regulatory processes and fostering a deeper understanding of biomolecular functions and mechanisms (Zhu et al., 2021; Dong et al., 2021).

In this study, we employed liquid chromatography-tandem mass spectrometry (LC-MS/MS) for full-spectrum metabolomics, combined with four-dimensional data-independent acquisition (4D-DIA) quantitative proteomics, to comprehensively assess plasma samples from patients with severe acne before and after isotretinoin treatment, as well as from healthy controls. Our aim was to elucidate the effects of severe acne and isotretinoin treatment on metabolites and proteins, with the goal of investigating the potential pathogenesis of severe acne and the mechanisms underlying isotretinoin’s action.

## Materials and methods

### Sample collection and processing

This study was approved by the Ethics Committee of Guangzhou Dermatology Hospital (approval number: gzsp202408). Patient information, including age, gender, and body mass index (BMI), was recorded. Disease severity was assessed using the Global Acne Grading System (GAGS) score, with a score ≥31 indicating severe acne (Bernardis et al., 2020). Exclusion criteria included pregnancy, breastfeeding, planning pregnancy in women and recent use (within the past 3 months) of oral isotretinoin or hormonal medications. Oral isotretinoin (Chongqing Huabang Pharmaceutical Co., Ltd., National Drug Standard approval number H20113060, dosage: 10 mg) was administered at a dosage of 20 mg daily (Dhaked et al., 2016). The control group comprised 20 healthy individuals matched for age and gender. Blood samples were collected into 5 mL EDTA-coated vacuum tubes, centrifuged at 3000 rpm for 10 min to isolate plasma, and stored in 200 μL aliquots at −80°C.

### Main reagents

Methanol, acetonitrile, isopropanol, and methyl tert-butyl ether (MTBE) were purchased from Merck. Ammonium formate and dichloromethane (DCM) were sourced from Fisher, while ammonia solution and iodoacetamide were obtained from Aladdin. Standards were purchased from BioBioPha/Sigma-Aldrich. Main reagents used in plasma proteomics were consistent with those described by Cao et al. (2023).

### LC/MS full-spectrum metabolomics analysis

Data Collection Instrumentation System: The system primarily consists of Ultra Performance Liquid Chromatography (UPLC) (ExionLC AD, https://sciex.com.cn/) and Tandem Mass Spectrometry (MS/MS) (QTRAP^®^, https://sciex.com.cn/).

Hydrophilic Substances: Plasma samples were thawed at 4°C. A 50 μL sample was mixed with 300 μL of a 20% acetonitrile/methanol internal standard extraction solution, followed by centrifugation at 4°C for 10 min. The supernatant (200 μL) was collected and centrifuged again for 3 min. The supernatant (180 μL) was transferred for LC-MS analysis. The detailed LC-MS analysis procedures were consistent with those described by Wang et al. (2024c) and Ji et al. (2024).

Hydrophobic Substances: Plasma samples were thawed at 4°C. A 50 μL sample was combined with 1 mL of internal standard lipid extraction solution, followed by 200 μL of water and centrifugation. The supernatant (200 μL) was mixed with 200 μL of lipid reconstitution solution and centrifuged for 3 min. The supernatant was used for analysis. The lipid extraction solution and lipid reconstitution solution as described by Zhang et al. (2023a). The chromatographic column, gradients and mass spectrum conditions were as described by Liu et al. (2022). The CAD parameter was set to medium.

### 4D-DIA quantitative proteomics analysis

The extraction, digestion and cleanup of serum protein were as described by Wang et al. (2024b). LC-MS/MS was used to perform 4D-DIA quantitative proteomic analysis of serum, as described by Wang et al. (2024c). Data acquisition was performed using the timsTOF Pro2 mass spectrometer in ddaPASEF mode. The specific parameters as described by Huang et al. (2024).

### Bioinformatics and statistical analysis

Metabolite identification was conducted using the in-house targeted reference database, MWDB (Metware Database). Quantification was carried out in multiple reaction monitoring (MRM) mode on a triple quadrupole mass spectrometer. Unsupervised principal component analysis (PCA) was conducted with the prcomp function in R (www.r-project.org). Hierarchical clustering analysis (HCA) was performed to assess metabolite accumulation patterns across different samples were generated using the Complex Heatmap package in R. Differentially expressed metabolites (DEMs) were determined by criteria including VIP >1 and p-value <0.05. VIP values were obtained from the Orthogonal Partial Least Squares Discriminant Analysis (OPLS-DA) results, which were generated using the R package MetaboAnalystR. To prevent overfitting, a permutation test with 200 iterations was conducted. Metabolites were annotated using the KEGG compound database (http://www.kegg.jp/kegg/compound/) and then mapped onto the KEGG pathway database (http://www.kegg.jp/kegg/pathway.html).

The MS raw data were processed using DIA-NN (v1.8.1) through a library-free approach. The database used was Uniprot_proteomeUP000005640_human_20230504.fasta, containing 82,492 sequences. Parameters were set to predict a spectral library constructed with deep learning, and MBR was used to construct a spectral library from DIA data for the purpose of protein quantification. The false discovery rate (FDR) was controlled at 1% for both the precursor ion and protein levels. Differentially expressed proteins (DEPs) were defined as those with FC > 1.5 or FC < 0.6667 and a p-value <0.05 (Chen et al., 2020). Differentially identified proteins were functionally annotated using Gene Ontology (GO) classifications (http://geneontology.org/) and Kyoto Encyclopedia of Genes and Genomes (KEGG) pathways (https://www.genome.jp/kegg/).

## Results

### Characteristics of study participants

**FIGURE 1:**
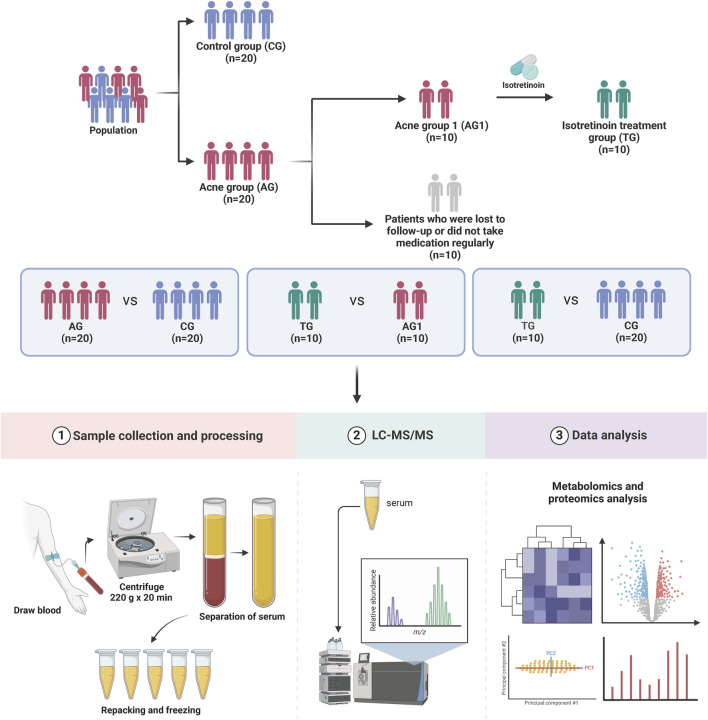
Overview of the study design and patient group allocation.

A total of 20 patients with severe acne (acne group, AG) and 20 healthy controls (control group, CG) were recruited for the study. No statistically significant differences were observed between the two groups regarding age and gender ratio, and BMI (p > 0.05). Among them, 10 patients (acne group 1, AG1) underwent regular isotretinoin treatment for 12 weeks (treatment group, TG) and were followed up with blood sample collection, with all of their acne symptoms showed significant improvement (p < 0.001). The main reasons for loss to follow-up included seeking follow-up care at local hospitals, non-adherence to the prescribed regimen, and the use of concomitant medications during the treatment period. Figure 1 illustrates the grouping and experimental procedures, while Table 1 summarizes the age, gender, BMI, and GAGS scores for each group.

**TABLE 1:** Cohort characteristics.

Characteristics	AG (n = 20)	CG (n = 20)	Statistic (AG vs. CG)	p-value (AG vs. CG)	AG1 (n = 10)	TG (n = 10)	Statistic (AG1 vs. TG)	p-value (AG1 vs. TG)
Age (Mean ± Standard Deviation, years)	22.55 ± 5.12	22.80 ± 2.38	t = −0.20	0.85	24.00 ± 6.55		—	—
Gender							
Male	10	10	—	—	4		—	
Female	10	10	—	—	6		—	
BMI (Mean ± Standard Deviation, kg/m²)	24.64 ± 4.07	23.73 ± 2.94	z = −0.92	0.36	23.62 ± 3.82	23.56 ± 3.27	t = −0.17	0.87
GAGS (Mean ± Standard Deviation)	34.95 ± 2.16	—	—	—	35.70 ± 2.00	10.80 ± 3.43	t = −21.85	<0.001

### Plasma metabolite changes and pathway analysis

**FIGURE 2:**
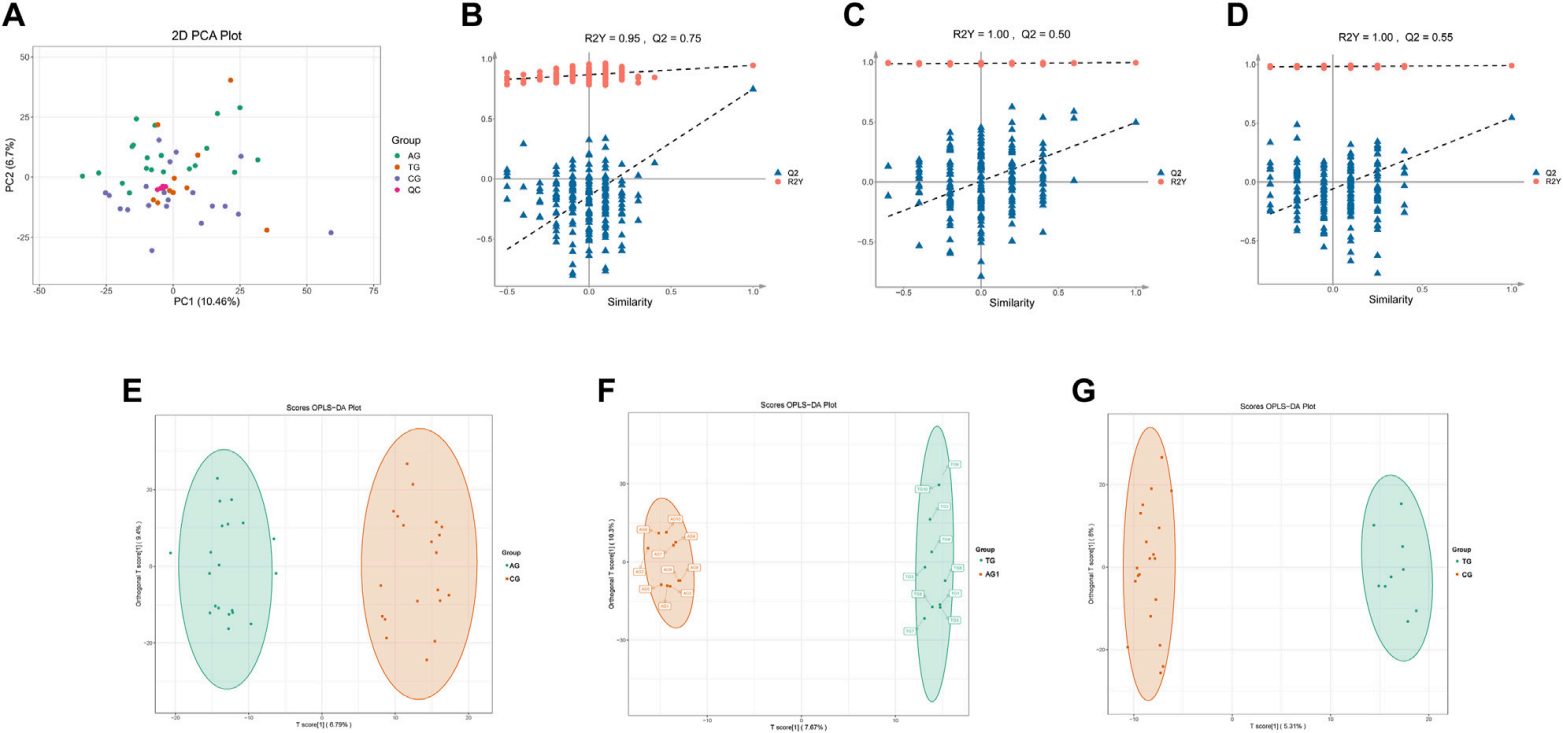
PCA plots for DEMs of samples from AG, TG, CG and QC **(A)**. Q2 and R2Y of the OPLS-DA model between the groups AG and CG **(B)**, TG and AG1 **(C)**, TG and CG **(D)**. OPLS-DA score plots between the groups AG and CG **(E)**, TG and AG1 **(F)**, TG and CG **(G)**.

**FIGURE 3:**
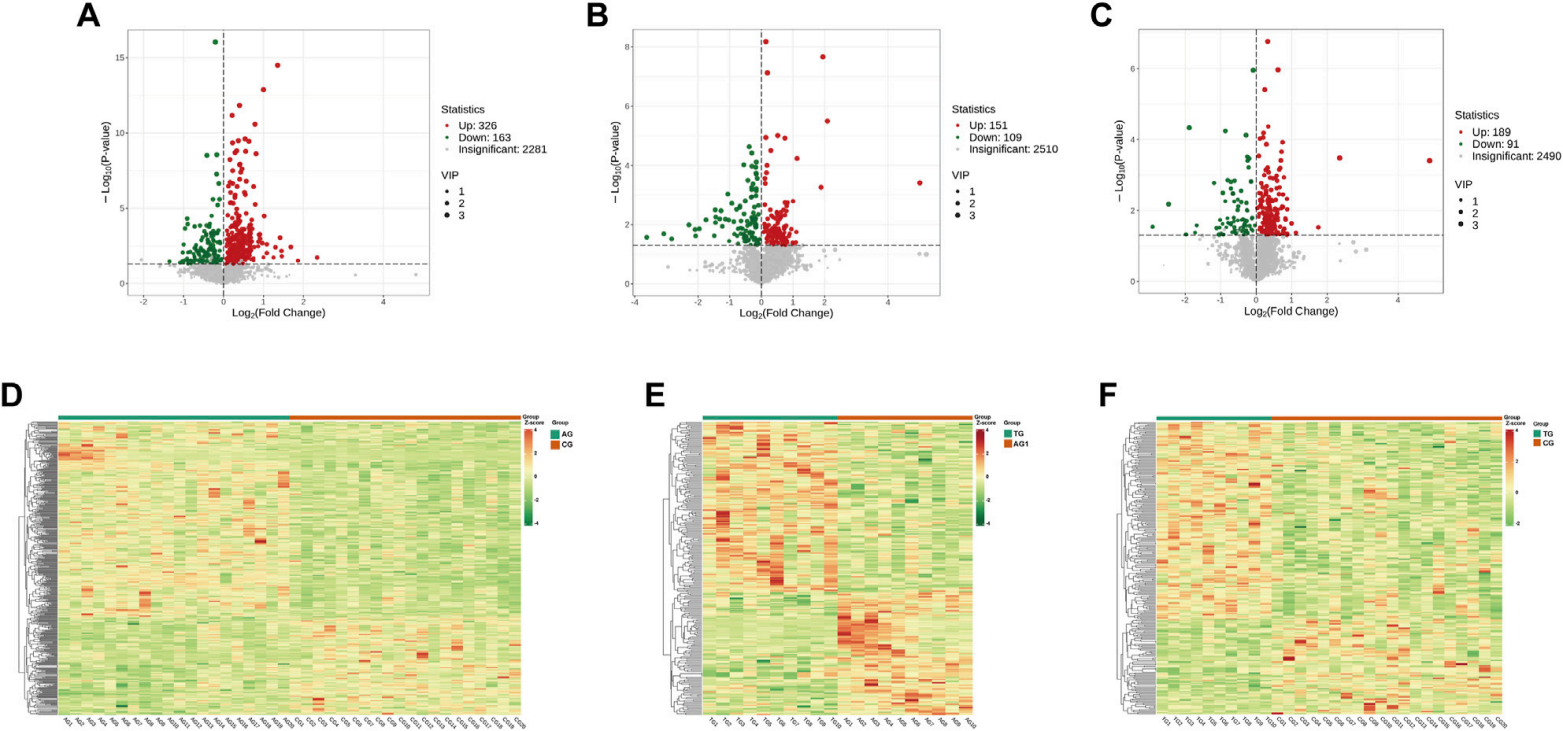
Volcano maps for DEMs between the groups AG and CG **(A)**, TG and AG1 **(B)**, TG and CG **(C)**. Clustering heat maps for DEMs between the groups AG and CG **(D)**, TG and AG1 **(E)**, TG and CG **(F)**.

The quality control (QC) samples, represented by blue spots, clustered tightly in the principal component analysis (PCA) plot (Figure 2A), suggesting the system exhibits good stability and is suitable for metabolomics analysis of the samples. Orthogonal projections to latent structures-discriminant analysis (OPLS-DA) models were constructed to evaluate the performance of model using *R* ^2^ (goodness of fit) and Q^2^ (predictive ability) values (n = 200), validating the accuracy of the OPLS-DA model (Figures 2B–D). The OPLS-DA model revealed significant separation in the metabolic profiles of the AG and CG groups, TG and AG1 groups, TG and CG groups (Figures 2E–G), indicating that both severe acne and isotretinoin treatment result in significant changes in plasma metabolic features. Volcano maps (Figures 3A–C) and hierarchical clustering heatmaps (Figures 3D–F) visually displayed the differences in metabolites among the groups.

A total of 2,281 metabolites were identified in AG and CG groups (Figure 3A). Among these, 489 DEMs were identified, mainly including glycerophospholipids (GPs), amino acids and their metabolites, glycerolipids, sphingolipids, organic acids, and their derivatives. When compared to CG group, 326 DEMs were upregulated in AG group. The upregulated Lipid DEMs mainly consisted of triglycerides (TG, 5.2%), such as TG (15:0_17:0_18:0), ceramides (Cer, 4.2%), lysophosphatidylcholine (LPC, 3.6%), diglycerides (DG, 3.0%), and sphingolipids (3.0%). Conversely, 163 DEMs were downregulated in AG group. The downregulated lipid DEMs mainly consisted of other triglycerides (10.4%), such as TG (12:0_16:0_22:6), phosphatidylethanolamine (6.0%), phosphatidylcholine (PC, 3.6%), free fatty acids (FFAs, 3.6%), and phosphatidylinositol (PI, 3%). KEGG analysis revealed that the DEMs in AG and CG groups were mainly associated with pathways such as tryptophan metabolism, thyroid hormone signaling pathway, sphingolipid signaling pathway, and inositol phosphate metabolism.

**FIGURE 4:**
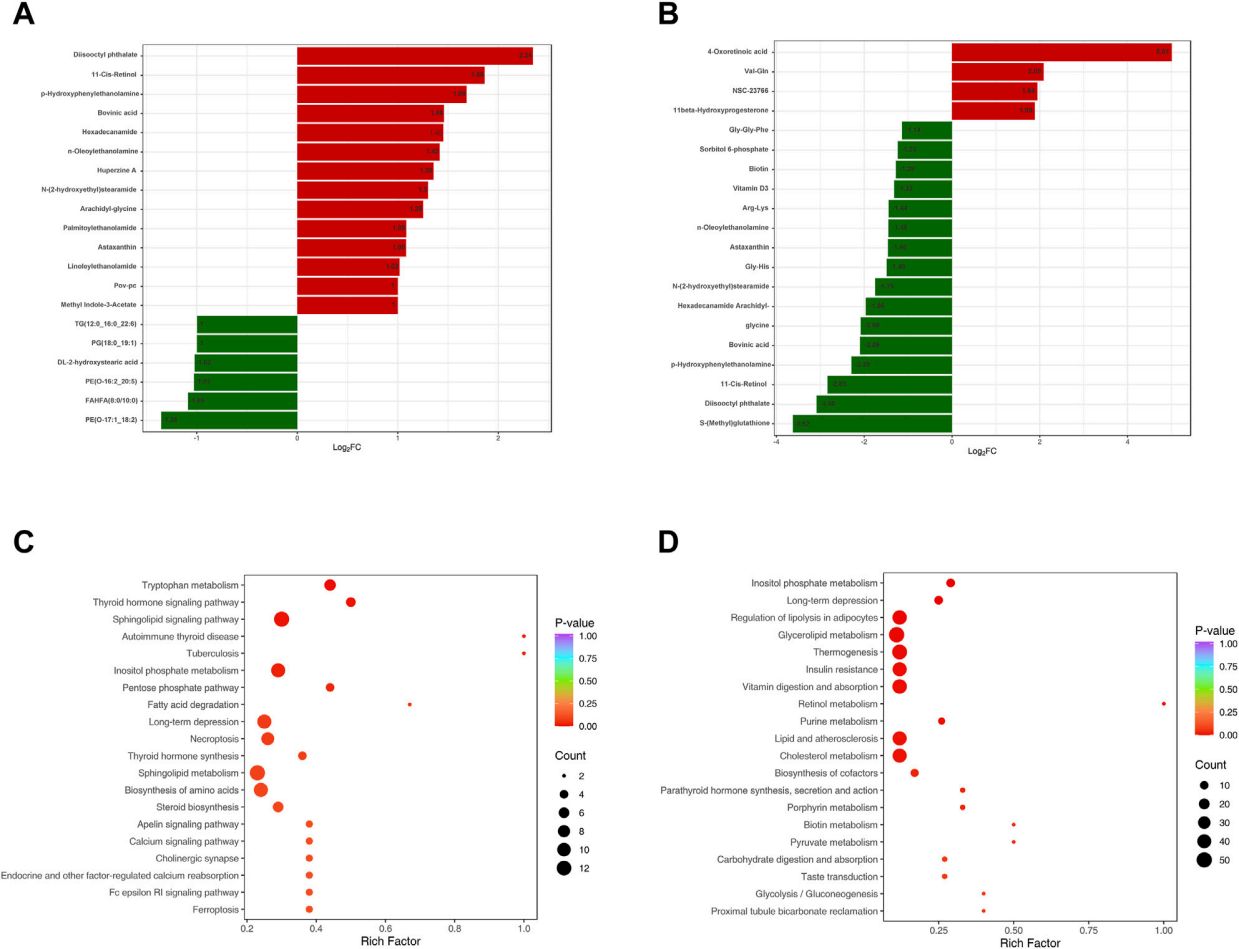
The top 20 DEMs between groups AG and CG **(A)**, TG and AG1 **(B)**. The top 20 KEGG pathways enriched with DEMs between groups AG and CG **(C)**, TG and AG1 **(D)**.

A total of 2,510 metabolites and 260 DEMs were identified in TG and AG1 groups (Figure 3B), mainly including glycerolipids, GPs, amino acids and their metabolites, phenols and their derivatives, and amines. Compared to AG1 group, 151 DEMs were upregulated in TG group. The lipid DEMs mainly consisted of triglycerides (22.5%), such as TG (17:1_18:2_18:3), DG (7.9%), Cer (5.2%), PC (3.9%), and LPC (3.3%). The TG group had 109 downregulated metabolites with fewer lipid DEMs, including other triglycerides (5.5%) such as TG(15:0_17:0_18:0), acylcarnitines (ACs, 5.5%), and monoglycerides (MGs, 2.7%). KEGG analysis revealed that the DEMs in TG and AG1 groups were mainly associated with pathways such as inositol phosphate metabolism, regulation of lipolysis in adipocytes, glycerolipid metabolism, and thermogenesis. Bar charts show the top 20 metabolites ranked by log_2_|FC| in each group (Figures 4A,B), while bubble plots illustrate the top 20 enriched KEGG pathways (Figures 4C,D).

After treatment, the TG group showed 280 DEMs, including 189 upregulated and 91 downregulated metabolites, compared with the CG group (Figure 3C). The major categories of these DEMs were similar to those observed between AG and CG groups, but the number was significantly reduced, indicating that isotretinoin had a corrective effect on the overall metabolic abnormalities in the plasma of patients with severe acne. A total of 126 DEMs exhibited a trend towards normalization after isotretinoin treatment, with 94 DEMs returning to levels indistinguishable from those in G group. Among these, 78 DEMs were upregulated in AG group and downregulated after isotretinoin treatment, mainly including alcohols and amines (15.3%), fatty acyls (12.8%), organic acids and their metabolites (11.5%), amino acids and their derivatives (10.2%), and glycerolipids (10.2%). There were 27 lipid metabolites, such as bovinic acid and n-oleoylethanolamine. Additionally, 48 DEMs were downregulated in AG group and upregulated after isotretinoin treatment, mainly including phenols and their derivatives (20.8%), organic acids and their derivatives (20.8%), and GPs (14.5%). These included 14 lipid metabolites, such as LPS(20:1), Cer(d16:1/23:0), and TG (17:1_18:2_18:3). Based on the standards of FC > 1.5 and FC < 0.6667, Table 2 was lists the 21 DEMs that exhibited both significant abnormalities and correction trends. Further details of the DEMs are shown in Supplementary Table S1.

**TABLE 2:** DEMs with significant abnormalities and correction trends.

Index	Compounds	Trend (AG vs. CG)	FC (AG vs. CG)	p-value (AG vs. CG)	Trend (TG vs. AG1)	FC (TG vs. AG1)	p-value (TG vs. AG1)
MEDP2706	Diisooctyl phthalate	up	5.08	0.019	down	0.12	0.020
MEDP0408	11-Cis-Retinol	up	3.64	0.031	down	0.14	0.030
MEDP2010	p-Hydroxyphenylethanolamine	up	3.21	0.004	down	0.20	0.010
MEDP2226	Bovinic acid	up	2.75	0.016	down	0.23	0.015
MEDP1131	Arachidyl-glycine	up	2.38	0.019	down	0.24	0.024
MEDP0513	Hexadecanamide	up	2.74	0.007	down	0.26	0.014
MEDP1040	N-(2-hydroxyethyl)stearamide	up	2.46	0.004	down	0.30	0.007
MEDP1744	Astaxanthin	up	2.12	0.002	down	0.36	0.003
MEDP2810	n-Oleoylethanolamine	up	2.67	0.001	down	0.37	0.009
MEDN0241	Vitamin D3	up	1.96	0.002	down	0.40	0.006
MEDP0143	Biotin	up	1.88	0.001	down	0.41	0.003
MEDP1670	Gly-Gly-Phe	up	1.61	0.006	down	0.45	0.007
MEDP1795	Palmitoylethanolamide (PEA)	up	1.88	0.001	down	0.49	0.007
MEDP2705	Hexadecanal	up	1.68	0.020	down	0.49	0.036
MEDP0277	Methyl Indole-3-Acetate	up	2.00	0.000	down	0.62	0.003
MEDP2345	His-Val	up	1.60	0.000	down	0.66	0.014
MEDP2285	NSC-23766	down	0.67	0.007	up	3.85	0.000
MEDN0278	2,6-Diaminopimelic acid	down	0.61	0.000	up	2.18	0.000
LIPID-P-1139	TG(17:1_18:2_18:3)	down	0.66	0.026	up	2.13	0.040
MEDN2386	LPS(20:1)	down	0.66	0.000	up	1.67	0.002
LIPID-P-0130	Cer(d16:1/23:0)	down	0.62	0.006	up	1.64	0.014

### Plasma protein changes and pathway analysis

**FIGURE 5:**
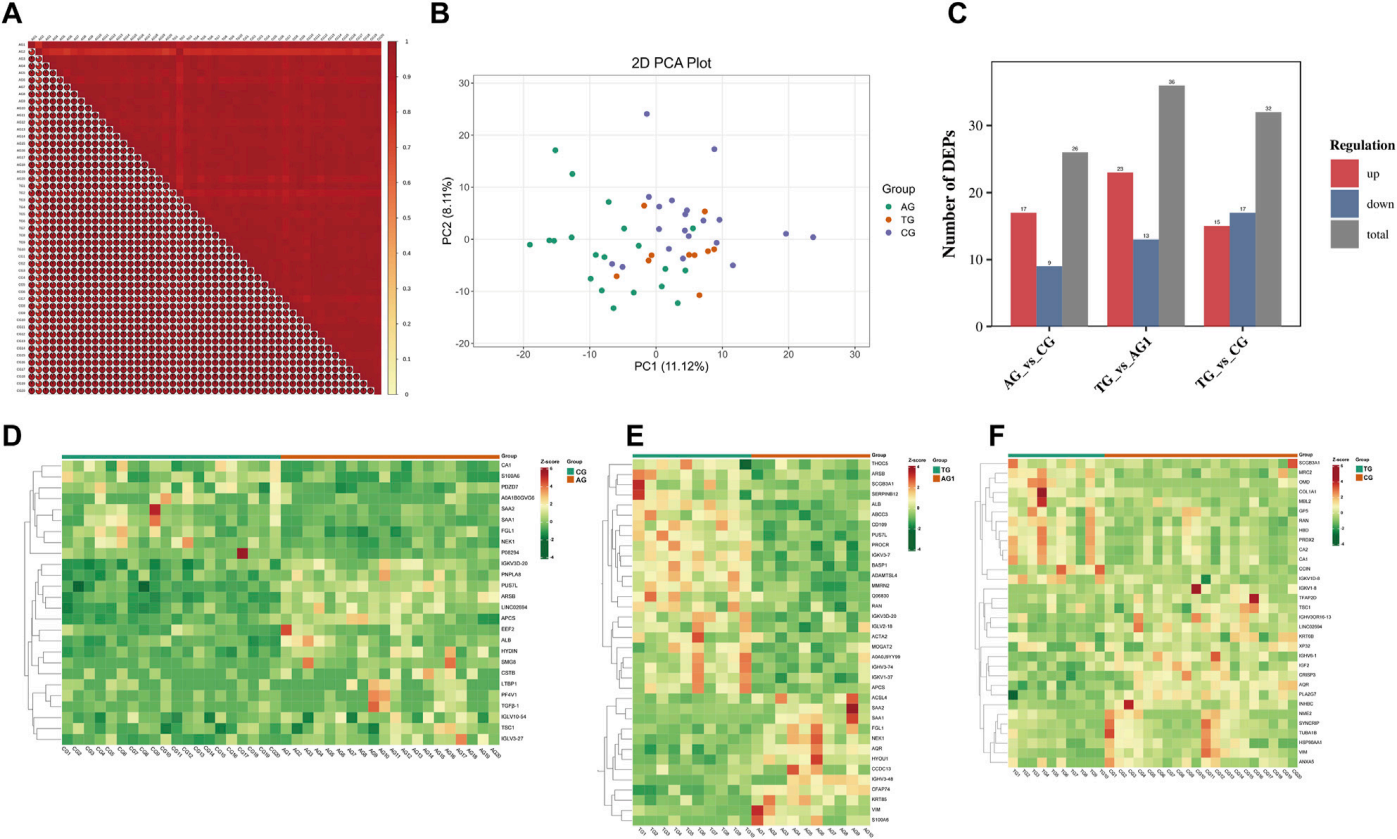
**(A)** The horizontal and vertical axes represent sample names, with colors ranging from red to yellow indicating a transition from high to low correlation. The size of the fan-shaped area and the numbers represent the magnitude of the correlation coefficient. **(B)** PCA plot of DEPs between the groups AG, CG and TG. **(C)** The number of DEPs between the groups. Clustering heat maps for DEPs between groups AG and CG **(D)**, TG and AG1 **(E)**, TG and CG **(F)**.

The protein abundance in the samples within the groups showed a Pearson correlation coefficient greater than 0.7 (Figure 5A), indicating strong consistency in the samples of each group and high reliability of DEPs. The PCA plot (Figure 5B) highlighted significant differences between the groups. A total of 23 DEPs were identified in AG and CG groups, with 17 upregulated and six downregulated in AG group. In contrast, 36 DEPs were identified between the TG and AG1 groups, with 23 upregulated and 13 downregulated in TG group. The bar chart shows the number of DEPs between the groups (Figure 5C). Further details of the DEPs are provided in the heatmaps (Figures 5D–F) and Supplementary Table S2.

**FIGURE 6:**
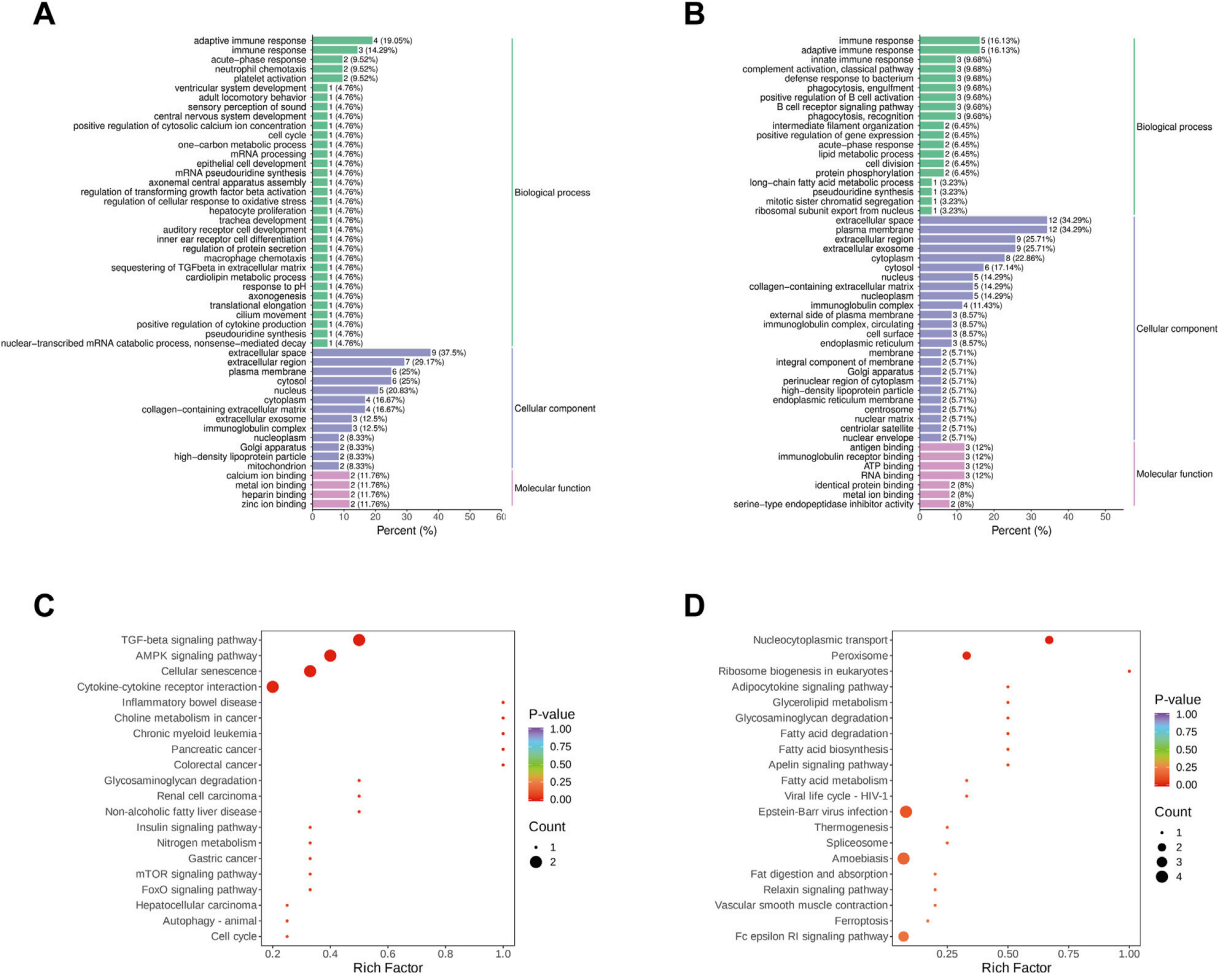
Go analysis for DEPs between groups AG and CG **(A)**, TG and AG1 **(B)**. KEGG enrichment analysis for DEPs between groups AG and CG **(C)**, TG and AG1 **(D)**.

GO analysis revealed that DEPs in both AG and CG, TG and AG1 groups were enriched in multiple biological processes (BP), cellular components (CC), and molecular functions (MF) (Figures 6A,B). The bar charts show that the DEPs in AG and CG group were involved in immune response, acute-phase response, neutrophil chemotaxis, platelet activation, and other biological processes. The main enriched cellular components encompassed the extracellular space, extracellular region, plasma membrane, nucleus and cytoplasm. These DEPs were associated with molecular functions, such as calcium ion binding, metal ion binding, heparin binding, and zinc ion binding. In contrast, the DEPs in TG vs. AG1 groups participated in BPs including immune response, complement activation(classical pathway), defense response to bacterium, positive regulation of B cell activation, and B cell receptor signaling pathway. The abundant CCs in this comparison encompassed the extracellular space, plasma membrane, extracellular region, extracellular exosome, and cytoplasm. The associated MFs included antigen binding, immunoglobulin receptor binding, ATP binding and RNA binding.

KEGG analysis indicated that the DEPs in AG vs. CG groups were primarily related to the TGF-β signaling pathway, AMPK signaling pathway, cellular senescence, cytokine-cytokine receptor interaction, and inflammatory bowel disease. Notably, the upregulation of the DEPs shuch as transforming growth factor-β 1 (TGF-β1) and tuberous sclerosis complex 1 (TSC1) participated in several inflammation-related pathways, including MAPK, PI3K-Akt, mTOR, FoxO, AMPK, Hippo, and TGF-β. Additionally, TGF-β1 associated with Th17 cell differentiation. In TG vs. AG1 groups, the DEPs were primarily related to nucleocytoplasmic transport, peroxisome, ribosome biogenesis in eukaryotes, and adipocytokine signaling pathways (Figures 6C,D).

### Combination of metabolics and proteomics

**FIGURE 7:**
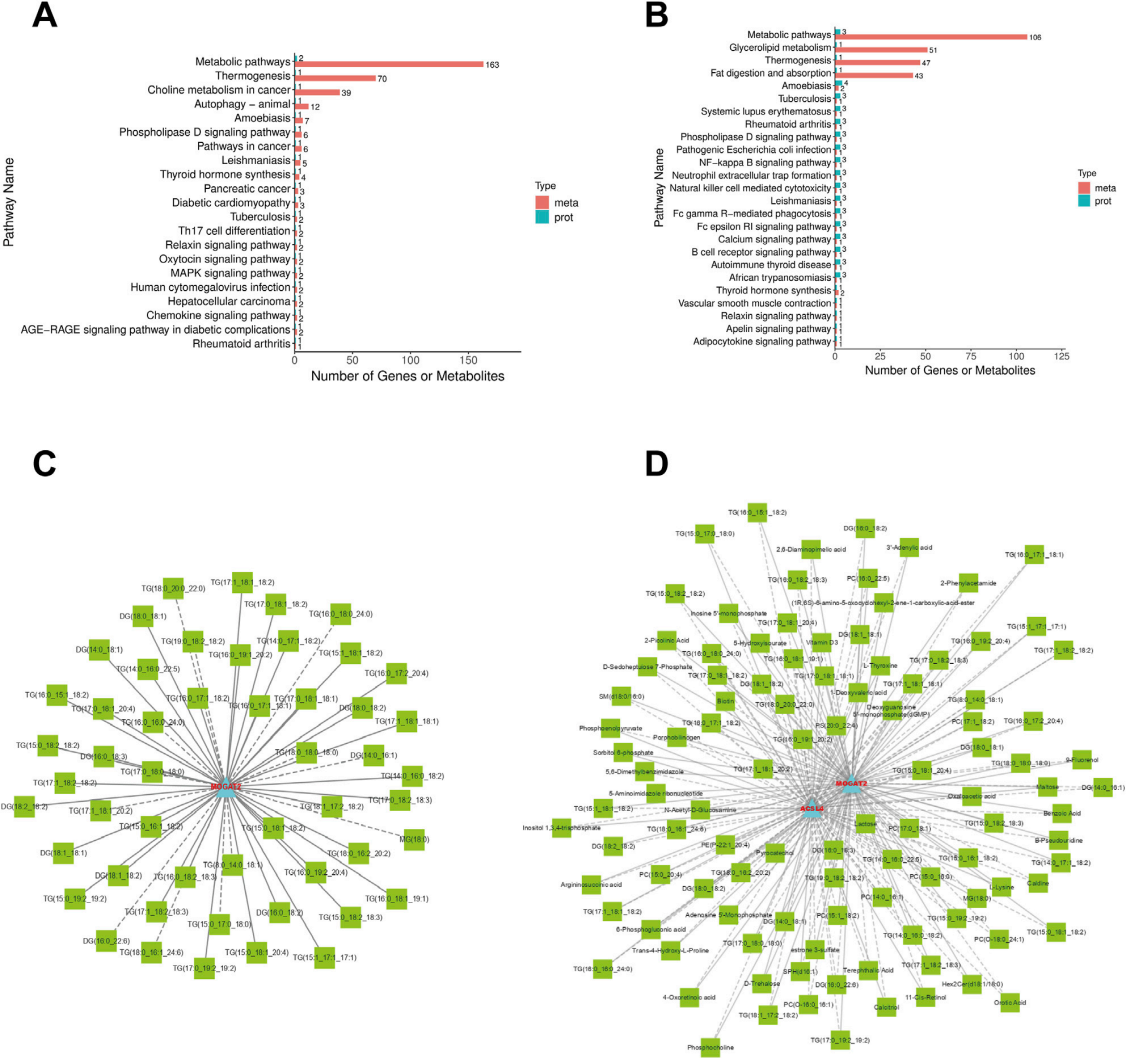
KEGG enrichment analysis for DEMs and DEPs between groups AG and TG **(A)**, TG and AG1 **(B)**. Correlation Network Diagram of glycerolipid metabolism **(C)** and metabolic pathways **(D)**: The diagram is constructed based on the correlation results of DEPs and DEMs that meet the following criteria: the absolute value of the Pearson correlation coefficient is greater than 0.8, p-value <0.05, FC > 1.5 or FC < 0.6667. DEMs are represented by green squares, and DEPs are represented by blue triangles. Solid lines indicate positive correlations, while dashed lines indicate negative correlations.

KEGG analysis revealed that the DEMs and DEPs in AG vs. CG groups, TG vs. AG1 groups, were collectively enriched in metabolic pathways, glycerolipid metabolism, thermogenesis, phospholipase D signaling pathway, and thyroid hormone synthesis (Figures 7A,B). Expression correlation analysis further indicated that within the glycerolipid metabolism (Figure 7C) and metabolic pathways (Figure 7D), monoacylglycerol O-acyltransferase 2 (MOGAT2) was upregulated in TG group compared to AG1 group, whereas acyl-CoA synthetase long-chain family member 4 (ACSL4) was downregulated. Those alterations were closely associated with the changes in various DEMs, including inositol 1,3,4-trisphosphate (Ins(1,3,4)P3), triglycerides, DG, MG, PC, sphingomyelins (SM), estrone 3-sulfate, and other.

## Discussion

In this study, we collected plasma samples from patients with severe acne before and after isotretinoin treatment, as well as from normal controls. Utilizing full-spectrum metabolomics and 4D-DIA quantitative proteomics analysis, we investigated the potential pathogenesis of severe acne and the mechanisms underlying isotretinoin’s efficacy for the first time.

### Lipid metabolism

Previous studies have shown significant alterations in lipid metabolism in acne patients, with elevated serum levels of total cholesterol, low-density lipoprotein (LDL), high-density lipoprotein (HDL), and triglycerides compared to healthy individuals (Sobhan et al., 2020; Romańska-Gocka et al., 2018). Furthermore, studies in rabbits with acne (Wu et al., 2024; Ou-Yang et al., 2022) have identified differential skin lipid metabolites, primarily consisting of Cer, phosphatidylethanolamine, and PC, with isotretinoin treatment reducing skin lipids levels and normalizing various skin lipid metabolites. Our results demonstrated that plasma triglycerides, Cer, LPC, DG, and sphingolipids in patients with severe acne were significantly upregulated compared to the normal group, while phosphatidylethanolamine, PC, FFAs, and PI were significantly downregulated. Consistent with previous studies (Zhang D. et al., 2023; Chen et al., 2021; Zhou et al., 2020), we also identified the significant role of sphingolipid metabolism and sphingolipid signaling pathways in acne, with differential sphingolipid metabolites included SM, sphingosines, and glycosphingolipids being upregulated in the plasma of patients with severe acne.

After isotretinoin treatment, multiple triglycerides, DG, Cer, PC, and LPC were significantly elevated, particularly triglycerides, which aligns with the side effect of blood lipid elevation (Costa et al., 2018). However, isotretinoin corrected 41 lipid metabolites that were abnormal in patients with severe acne, such as bovinic acid, n-oleoylethanolamine, LPS(20:1), Cer(d16:1/23:0), and TG (17:1_18:2_18:3). Insulin resistance, which increases sebum secretion, alters androgen levels, and exacerbates inflammation, contributes to acne development (Bungau et al., 2023). Our findings suggest that isotretinoin reduces the abundance of specific triglycerides, such as TG (15:0_17:0_18:0), TG (17:0_18:0_18:0), and TG (18:0_18:0_18:0), which are enriched in the insulin resistance pathway and may contribute to improved insulin sensitivity. Notably, isotretinoin upregulated protein MOGAT2, which is associated with the alterations in various TG and DG. MOGAT2, an important membrane-bound acyltransferase involved in the synthesis of DG and triglycerides, is associated with hyperlipidemia, obesity, and insulin resistance (Abdel-Magid, 2014; Yang and Nickels, 2015). We speculate that the elevation of blood lipids and the correction of some abnormal lipids observed during isotretinoin treatment may be mediated by the upregulation of MOGAT2. Pathway enrichment analysis suggests that isotretinoin regulates blood lipid through pathways such as regulation of glycerolipid metabolism, lipolysis in adipocytes, cholesterol metabolism, and fatty acid metabolism.

### Inositol phosphate metabolism

Given the significant enrichment of DEMs and DEPs, our study focused on the inositol phosphate metabolism pathway, which has been implicated in multiple diseases, particularly metabolic disorders such as insulin resistance and polycystic ovary syndrome (Chatree et al., 2020; Unfer et al., 2020). Specifically, Ins(1,3,4)P3 was upregulated in patients with severe acne and returned to normal level following isotretinoin treatment. Despite limited recent research on Ins(1,3,4)P3, it plays a crucial role in regulating calcium ion efflux and storage, thereby maintaining cellular calcium signaling (Tio et al., 1991; King et al., 1990). Calcium signaling is essential for regulating sebaceous gland secretion, influencing immune cells activation, and the release of pro-inflammatory cytokines (Katsuta et al., 2005). Consequently, the upregulation of Ins(1,3,4)P3 may lead to activation of calcium signaling, exacerbating sebaceous gland secretion and local inflammation. Furthermore, as an intermediate in inositol phosphate metabolism, Ins(1,3,4)P3 may indirectly activate PI3K/AKT and other inflammatory signaling pathways associated with phospholipids (Tan et al., 2015). Therefore, the upregulation of Ins(1,3,4)P3 and disruption of inositol phosphate metabolism may be closely linked to severe acne, with isotretinoin potentially exerting therapeutic effects by normalizing plasma Ins(1,3,4)P3 levels.

### Hormonal metabolism

Isotretinoin may partially alleviate acne by addressing thyroid hormonal metabolic disorders. Elevated thyroid hormone levels, specifically thyroxine (T4), have been shown to increase the metabolic rate and sebaceous gland activity, leading to increased sebum production (Clayton et al., 2020; Bungau et al., 2024). Additionally, T4 can synergize with androgens to trigger acne (Clayton et al., 2020; Bungau et al., 2024). Our study observed elevated T4 levels in the plasma of AG group, which normalized after isotretinoin treatment. This suggestes that isotretinoin may exert its therapeutic effect in severe acne by downregulating T4 in the thyroid hormone synthesis pathway. However, excessive downregulation could lead to thyroid dysfunction, as previous studies have linked isotretinoin treatment to hypothyroidism, potentially due to dosage and treatment duration (Salem et al., 2021).

Furthermore, our study indicated that isotretinoin might have the potential to suppress androgens, which play a crucial role in acne pathogenesis by promoting sebaceous cell proliferation, abnormal differentiation, pro-inflammatory cytokine secretion, and inhibiting sebaceous cell autophagy (Bungau et al., 2023; Elshazly and Gewirtz, 2023). We observed a trend toward upregulation of androstenediol in group AG, which showed a decreasing tendency after isotretinoin treatment. Conversely, estrone 3-sulfate showed the opposite trend.

### Inflammatory responses

In this study, TGF-β1 was upregulated in the AG group compared to the CG group and showed strong associations with multiple inflammatory signaling pathways in the KEGG analysis. TGF-β1, a multifunctional cytokine, regulates cell proliferation and differentiation, immune response, and tissue repair (Huang et al., 2020; Wang H. et al., 2024). It participates in multiple inflammation-related signaling pathways, including TGF-β, MAPK, PI3K-Akt, mTOR, FoxO, AMPK, and Hippo (Song et al., 2020; Yadav et al., 2017; Ma et al., 2022). Overexpression of TGF-β1 may promote sebaceous gland proliferation and secretion, exacerbate abnormal keratinization in hair follicles, and intensify inflammatory responses. Additionally, upregulation of TGF-β1 promotes Th17 cell differentiation (Umeda et al., 2021), resulting in increased secretion of pro-inflammatory cytokines such as IL-17, which further exacerbates local inflammation. The TGF-β signaling pathway plays a crucial role in skin healing and tissue repair, particularly in collagen synthesis; how excessive activation may potentially lead to the formation of acne scars (Eremenko et al., 2024). Therefore, elevated TGF-β is essential in the progression of acne, and targeting it may provide novel therapeutic strategies for acne treatment, particularly in controlling inflammation and preventing scarring. In contrast, the upregulation of DEP TSC1 may have potential beneficial effects on acne, as it can inhibit mTORC1 (Bovari-Biri et al., 2023), whose excessive activation promotes sebum production,abnormal keratinization of sebaceous gland, and enhances the local inflammatory response by upregulating the NF-κB pathway and the release of pro-inflammatory cytokines (Melnik, 2021; Melnik and Zouboulis, 2013; Miao et al., 2023). We hypothesize that upregulation of TSC1 may serve as a self-protective mechanism in severe acne.

Furthermore, we found that isotretinoin downregulated ACSL4, an enzyme involved in lipid metabolism, that catalyzes the biosynthesis of arachidonoyl-CoA and polyunsaturated fatty acid-containing lipids, which are closely related to the changes of many metabolites. ACSL4 contributes to the execution of ferroptosis by triggering phospholipid peroxidation and promoting the accumulation of lipid peroxidation products. (Li et al., 2023; Ding et al., 2023; Zhang et al., 2022). Gao et al. showed that cancer-associated fibroblast-secreted exosomal miR-454-3p inhibits lipid metabolism and ferroptosis in breast cancer by targeting ACSL4 (Gao et al., 2024). Thus, the downregulation of ACSL4 may reduce sebaceous gland synthesis, inhibit ferroptosis-related cell damage, decrease arachidonic acid activation, and synthesis of pro-inflammatory cytokines, suggesting a potential therapeutic mechanism for isotretinoin in the treatment of severe acne.

### Amine and astaxanthin

Interestingly, we observed a significant upregulation of various protective amine metabolites, such as hexadecanamide, arachidyl-glycine, and palmitoylethanolamide, as well as astaxanthin in patients with severe acne. Following isotretinoin treatment, these metabolites were downregulated to normal group levels. Previous studies have indicated that these substances exertanti-inflammatory effects (Console-Bram et al., 2017; Deveci et al., 2020; Clayton et al., 2021; Lama et al., 2022; Bao et al., 2023), potentially regulating sebaceous gland secretion and suppressing the growth of Cutibacterium acnes. Astaxanthin demonstrates potent antioxidant and anti-inflammatory properties, as well as inhibiting ferroptosis (Zhang et al., 2024; Cai et al., 2022). We hypothesize that the compensatory upregulation of these protective metabolites aids in mitigating damage. After isotretinoin treatment, acne symptoms improved, and these DEMs were significantly downregulated to baseline levels.

## Conclusion

This study demonstrates that dysregulated blood lipids and hormone levels, along with abnormalities in inositol phosphate metabolism, sphingolipid signaling pathway, TGF-β signaling pathway, and Th17 cell differentiation, are closely associated with the progression of severe acne. Treatment with isotretinoin normalized the dysregulation of 94 metabolites. The therapeutic efficacy of isotretinoin in severe acne is attributed to its potential mechanisms, including the reduction of inflammation, regulation of lipid, inositol phosphate, and hormone metabolism, and alleviation of insulin resistance. The downregulation of ACSL4 may indicate a potential therapeutic mechanism for isotretinoin, whereas upregulation of MOGAT2 may mediate the elevation of blood lipids and the correction of some abnormal lipids. This research establishes a foundation for future investigations into the mechanisms underlying severe acne and the action of isotretinoin, offering novel insights into therapeutic targets, treatment optimization, and side effect reduction. Nevertheless, our study has certain limitations. This study was limited by a relatively small sample size and a high dropout rate, which may have introduced potential bias and impacted the reliability of the efficacy evaluation. Future studies with larger cohorts and experimental validation were considered necessary to improve the reliability of these findings. Besides, the changes, mechanisms, and functions of differential metabolites and proteins require further validation and investigation, and the associated metabolic and inflammatory pathways remain to be verified and explored.

## Data Availability

The data reported in this paper have been deposited in the OMIX, China National Center for Bioinformation/Beijing Institute of Genomics, Chinese Academy of Sciences (https://ngdc.cncb.ac.cn/omix, accession no. OMIX011173).
